# Evaluation of the safety and efficacy of skin penetration enhancer Putocrin®

**DOI:** 10.1111/jocd.16409

**Published:** 2024-06-12

**Authors:** Zongxiao Cen, Zhiyuan Chen, Ding Wang, Xueping Chen, Junyuan Chen

**Affiliations:** ^1^ Guangzhou Luanying Cosmetics Co., Ltd Guangzhou China; ^2^ Centre for Biotech Big Data Research & Development Research Institute of Tsinghua Pearl River Delta China; ^3^ Vitargent (International) Biotechnology Limited Hong Kong China

**Keywords:** EpiKutis®, keratinocytes, pigskin‐Franz cell, Putocrin®, skin penetration

## Abstract

**Background:**

Substances that can efficiently enhance skin penetration while exerting no adverse effect are useful for drug and cosmetics formulation.

**Objective:**

To investigate the safety and enhance skin penetration efficacy of Putocrin®, a combination containing 2% isosorbide dimethyl ether, 1% pentanediol, and 0.5% inositol.

**Methods:**

An in vitro keratinocyte cell assay using 3‐(4,5‐dimethylthiazolyl‐2)‐2,5 diphenyltetrazolium bromide (MTT), and an in vitro EpiKutis® skin study adopted hematoxylin and eosin staining, immunostaining, and liquid chromatography–mass spectrometry (LC–MS) analysis were carried out to investigate the safety of Putocrin®. A pigskin‐Franz cell system experiment applied high‐performance liquid chromatography (HPLC) to compare the skin penetration efficiency of fluorescein isothiocyanate (Fitc)‐labeled tranexamic acid with or without the assistance of Putocrin®. The safety and efficacy of Putocrin® was further evaluated on zebrafish embryos.

**Results:**

The MTT assay showed that Putocrin® at concentration ≤2.5% did not significantly affect cell viability. The in vitro EpiKutis® skin study revealed that 2.5% Putocrin® did not affect skin morphology, filaggrin content, ceramide/protein, or fatty acid/protein ratios, but significantly increased loricrin content by 86.00% (*p* < 0.001). The pigskin‐Franz cell penetration experiment demonstrated that Fitc‐labeled tranexamic acid could barely penetrate the skin (with penetration rate of 1.121%), while Putocrin® significantly enhanced the penetration rate up to 83.983%, which was close to unlabeled tranexamic acid (90.013%). The zebrafish embryo study showed that 2.5% Putocrin® did not exert observable toxicity and obviously assisted the skin penetration of Fitc‐labeled tranexamic acid into fish embryos. These results indicate the strong enhancing skin penetration potency of Putocrin®.

**Conclusion:**

This study demonstrated the safety as well as the strong enhancing skin penetration potency of Putocrin® for cosmetics formulation use.

## 1 INTRODUCTION

Skin is the largest organ that interfaces with the environment and plays a vital role in protecting the body from excessive water loss from internal organs and limit xenobiotics and hazardous substances entering the body. The stratum corneum, the most superficial layer of the skin, is composed by corneocytes (dead keratin‐containing cells) surrounded with lipid matrix to form brick‐and‐mortar structure with remarkable skin barrier function.^1^ The cornified envelope surrounding corneocytes makes it difficult for many substances to penetrate inside. The intercellular lipid domain that is constituted by ceramides, cholesterol, and free fatty acids in a roughly molar ratio of 1:1:1^2^ forms the most important pathway for substances to penetrate into the skin. Due to the lipophilic properties of the stratum corneum, hydrophilic substances penetrate poorly. To overcome the skin barrier and deliver active ingredients to the deeper skin layers like the epidermis or dermis, drug and cosmetics formulation scientists have devoted considerable efforts to identify effective skin penetration enhancers (SPEs).^3^, ^4^

SPEs are used in many topical cosmetics to promote the delivery of active ingredients into the skin. To evaluate the penetration efficiency of varying SPEs, a variety of methods has been explored ranging from in vitro cell and artificial skin, ex vivo mammal skin to in vivo human studies,^5^ of which in vitro skin^6^ and ex vivo skin^7^ were most widely applied in recent years. According to OECD TG 428, skin from many mammalian species, including humans, can be used for skin absorption studies.^8^ The OECD TG 428 method has been used to investigate the penetration activities of 56 cosmetic‐relevant chemicals with logP between−1 and 4, molecular weight between 100 and 500, and low volatility using ex vivo human skin, providing valuable information to better understand dermal delivery.^4^ A review of total 92 datasets collected from 30 published studies found the excised human skin model produced similar results as living humans.^7^ Further, the OECD TG 428 validation study demonstrated pig skin could be used as an alternative when human skin is not available.^6^

The working mechanisms of SPEs have been proposed into two categories.^3^, ^9^ The first is to increase the solubility of the stratum corneum by transiently changing the skin barrier solubility parameters, which consequently result in an improvement of the flux of the ingredients into the skin.^9^ Solvents such as propylene glycol, ethanol, and diethylene glycol monoethyl ether (commercially known as Transcutol®) were believed to work in this manner.^9^ The second mechanism is to change the order of structured lipids of the stratum corneum and cause an increase in the diffusion coefficient of the ingredients.^9^ For example, the emulsifier isopropyl myristate was considered to act in this way.^9^ However, due to the complexity of interactions between SPEs and the skin, and the interference of the vast diverse chemicals within a formulation, the mechanisms of action of SPEs are not well understood.^10^ One sure thing is that overcoming the skin barrier in an efficient, temporary, and safe manner remains a challenge as high permeation‐enhancing potency is often accompanied with toxicity and irritation.

Zebrafish shares up to 82% human disease‐related gene similarity,^11^ has been widely used as a model organism in recent decade, widely used for toxicity testing,^12^, ^13^ disease modeling,^14^ drug discovery,^15^ and also promoted as a dermatology model in recent years.^16^, ^17^

In this study, the safety, as well the enhancing skin penetration potency of a skin penetration‐enhancer named Putocrin® was investigated. Its toxicity was analyzed using an in vitro keratinocyte assay and an EpiKutis® skin study checking the skin morphology, filaggrin, and loricrin protein content changes as well as ceramide/protein and fatty acid/protein ratios. To determine the enhancing skin penetration activity, a pigskin‐Franz diffusion cell experiment was performed in compliant with OECD TG 428 using Fitc‐labeled tranexamic acid as example. The safety and efficacy of Putocrin® was further evaluated through zebrafish embryo study.

## 2 MATERIALS AND METHODS

### 2.1 Chemicals and reagents

The following reagents were used: Putocrin® containing 2% isosorbide dimethyl ether, 1% pentanediol, and 0.5% inositol provided by Guangzhou Luan Ying Cosmetics Co. Ltd, keratinocytes (batch number: EP230129), Fitc‐labeled tranexamic acid from Shanghai Apeptide Co. Ltd. (batch number: BX‐230831‐G1231), EpiKutis® (batch number: ES230602), KcGrowth culture medium and EpiGrowth culture medium from Guangdong Biocell Biotechnology, PBS from Solibo, WY14643, MTT and dimethyl sulfoxide (DMSO) from Sigma, isopropanol, xylene, methanol, lauryl sodium sulfate, sodium dihydrogen phosphate, and phosphoric acid from sinopharm, 4% paraformaldehyde tissue fixative from Biosharp, hematoxylin and eosin from Beyotime, triethylamine from Fuchen, anti‐loricrin (ab198994), anti‐filaggrin (ab198994) and anti‐human nuclear antigen (ab191181) primary antibodies, and goat anti‐rabbit (Alexa Fluor® 488, ab150077), and goat anti‐mouse (Alexa Fluor® 647, ab150117) secondary antibodies from Abcam.

### 2.2 Cytotoxicity test

Keratinocytes were incubated using KcGrowth culture medium in 96‐well plates in a CO_2_ incubator (150I, Thermo) at 37°C with 5% CO_2_ till 40%–60% cell laying rate. Putocrin® was diluted using culture medium and added to keratinocytes wells at 200 μL per well and replicated by triplicate per treatment. A blank control and toxicity positive control (10% DMSO) were included. After 24 h incubation, MTT of 0.5 mg/mL was added and incubated for 4 h in dark, and the OD value at 490 nm was read using microplate reader (BioTek, Epoch). Relative cell viability (%) was calculated as (averaged sample well OD – averaged adjusting well OD)/ (averaged solvent control well OD – averaged adjusting well OD) × 100%.

### 2.3 In vitro skin assay

Reconstructed human epidermal skin EpiKutis® (batch number: ES230602; Guangdong Biocell Biotechnology Co. Ltd) were cultured using EpiGrowth culture medium in 6‐well plates at 37°C with 5% CO_2_, 25 μL of 2.5% (v/v) Putocrin® solution evenly added on the surface of the skin. A blank control and positive control (50 μM of WY14643) were also included. After 24 h incubation at 27°C, the surface of the skin was washed with sterile PBS solution. The skin samples were fixed with 4% paraformaldehyde for 24 h, some of the sections were hematoxylin/eosin (H&E) stained and imaged using an orthography microscope (Olympus, BX5) to check the skin morphology, the other sections were processed for fluorescence immunostaining against filaggrin and loricrin and imaged using fluorescence microscope (Leica, DM2500) and integral optical density of fluorescence signal was analyzed with image‐Prof® Plus Image processing software. For lipid content analysis, skin samples were digested using proteinase K at 50°C for 2 h, the stratum corneum was picked out, rinsed in deionized water and sonicated in an ice water batch for 2 min, transferred to the methanol:chloroform = 1:2 solution and sonicated again for 2 min, followed by repeating the procedure twice. The stratum corneum was removed and the solution was dried under nitrogen gas flow, redissolved using isopropanol, centrifuged at 14 000 rpm for 10 min and supernatant was used for ceramide and fatty acid detection using LC–MS (Thermo Fisher Scientific, Q Exactive). The improvement rate was calculated as (sample group–blank control group)/ blank control group × 100%.

### 2.4 Skin penetration study using pigskin‐Franz cell system

A pigskin‐Franz cell system was used for skin penetration study. The back skin of 1‐month‐old suckling pig was stored at−20°C, which was thawed with deionized water at room temperature and rinsed repeatedly with PBS buffer. The pigskin‐Franz cell system was established using TK‐12D transdermal absorption diffuser (Shanghai Kaikai Trade Co., Ltd) (see Figure S1) for the detailed set up information). PBS of 7 mL was added into the receiving chamber, the pig skin was fixed between the supply chamber and the receiving chamber with stratum corneum side facing the supply chamber, then 1.5 mL of PBS was added to the sampling tube so that the dermal level of the skin was in close contact with the PBS receiving solution. After that, the electromagnetic stirrer was set to stir at the speed of 300 rpm, the water batch temperature was set 32 ± 1°C with no bubbles between water and skin was allowed. Sample of 300 μL was added to the surface of the skin and the supply chamber was sealed with a sealing membrane. Six replicates were set for each time point of each sample. At 1, 6, and 24 h after treatment initiation, 2 mL PBS receiving solution was extracted for high‐performance liquid chromatography (HPLC) analysis, and 2 mL of PBS was added back right after the extraction. At the end of 24 h treatment, the skin surface was cleaned by sucking PBS for five times and dried with a cotton swab, the permeated part of the skin was circumcised and fixed using 4% paraformaldehyde solution for at least 24 h, and then processed for sectioning and imaging using positive fluorescence microscope (Olympus) and analyzed with image‐Prof® Plus Image processing software. For the non‐permeated part of the skin, the skin surface was cleaned by sucking and blowing PBS repeatedly for five times with 1 mL of PBS each, and the PBS solution was collected for HPLC analysis. The residual part of the skin was cut along the edge of the diffusion chamber, cut into pieces and sonicated for 60 min using 5 mL of PBS, the supernatant was collected for HPLC analysis.

HPLC analysis was performed using Shimadzu 2030Plus equipped with C_18_ column, the mobile phase was composed of 0.23% sodium dodecyl sulfate solution (pH = 2.5): methanol = 60: 40, with flow rate of 1 mL/min and detection wavelength of 220 nm. Cumulative penetration was calculated according to equation (1). Diffusion percentage was calculated as total contents of the sample within receptor fluid/ initial loading volume of the sample in the diffusion chamber. Permeability coefficient Kp (penetration rate) was calculated through regression analysis between cumulative penetration (Y‐axis) against permeation time (X‐axis) to get the regression equation slope. Recovery rate was calculated as total contents (content of the skin surface + content in the skin + content of receptor fluid) / initial loading × 100%.

(1) $$Q=\left(C_{n}\times V+\sum C_{i}\times V_{0}\right)\left(i=1\cdots n-1\right)$$

In this formula *Q* is cumulative penetration; V is the volume of the receiving chamber, which is 8.5 mL; V_0_ is the volume of each sample collection, which is 2 mL; *C*_*i*_ is sample concentration is the receiving solution from the first to the *n*−1 sampling; C_n_ is sample concentration measured at the n^th^ sampling point.

### 2.5 Zebrafish embryo experiment

AB strain zebrafish colony maintenance and embryo collection was carried out as previously described.^13^ Healthy zebrafish embryos of 48 h post fertilization (hpf) were exposed to 2.5% Putocrin®, 1% Fitc‐labeled tranexamic acid, 2.5% Putocrin® together with 1% Fitc‐labeled tranexamic acid respectively at a density of 25 embryos in 5 mL solution. Normal embryos were used as blank control. After incubation at 28°C for 2 h, embryos were rinsed using water for three times and observed and imaged using a fluorescence microscope (Nikon DS‐Qi2).

### 2.6 Statistical analysis

The results were expressed as mean ± SEM. Comparisons between groups were performed using two‐tailed *t*‐test and Wilcoxon signed‐rank test, and *p* < 0.05 was considered as a significant difference.

## 3 RESULTS

### 3.1 Cytotoxicity of Putocrin® to keratinocytes

Keratinocyte cytotoxicity analysis aimed to identify the safety range of Putocrin®. Compared with the blank control, the cell viability of keratinocytes treated with 10% DMSO (toxicity positive control) was 0.88% ± 0.14%, indicating keratinocytes responded normally to toxicity. Based on the cell morphology and cell viability results (Figure 1), Putocrin® did not exhibit significant cytotoxicity with the concentration ≤2.5% (v/v).

**FIGURE 1:**
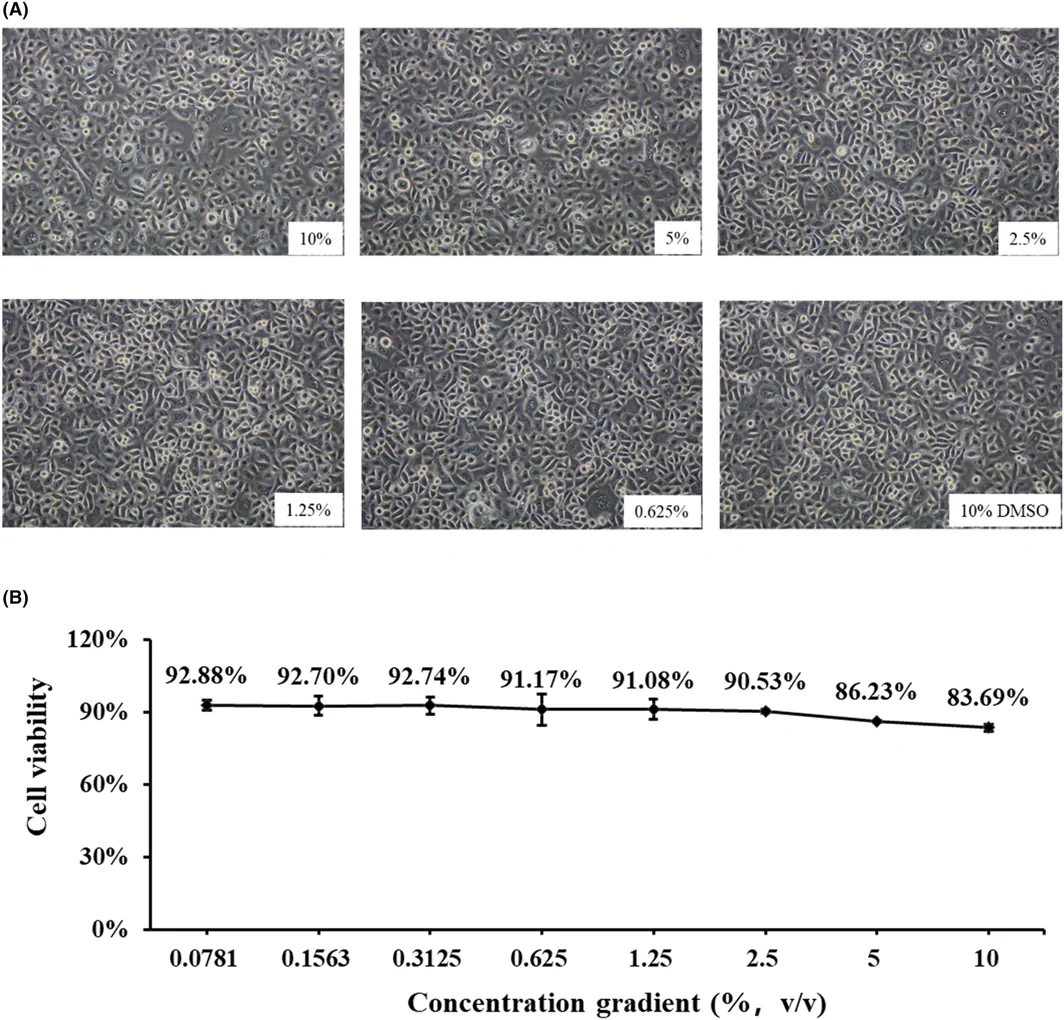
Cytotoxicity of Putocrin® to keratinocytes. Keratinocytes were pre‐incubated with Putocrin® (0.0781%‐10% gradations), 10% DMSO (toxicity positive control), and culture medium (blank control; data not shown) respectively for 24 h followed by cell viability analysis using MTT method. (A) Cell morphology at the end of the experiment. (B) Relative cell viability (%) was denoted as percentage difference (Mean ± SEM) over blank control.

### 3.2 Effects of Putocrin® to in vitro skin

EpiKutis® in vitro skin experiment was used to investigate the effects of Putocrin® to the skin barrier. The H&E staining (Figure 2A) showed that compared with the blank control, Putocrin® at 2.5% (v/v) had no significant effect on skin morphology, while the positive control WY14643 (50 μM) that known to positively regulate skin barrier functions, showed a significant increase in the number of living cells, demonstrating the responsiveness of the skin to the treatment. Immunostaining (Figure 2A–C) of skin barrier‐associated proteins filaggrin and loricrin revealed that compared with the blank control, WY14643 significantly (*p* < 0.01) increased the content of both filaggrin and loricrin, and Putocrin® of 2.5% (v/v) did not significantly change filaggrin content, while increasing loricrin content by 86.00% (*p* < 0.01). LC–MS analysis (Figure 2D,E) found out that compared with blank control, both ceramide/protein ratio and fatty acid/protein ratio of the stratum corneum significantly (*p* < 0.01) increased after WY14643 exposure, while kept stable (*p* > 0.05) after 2.5% Putocrin® treatment. All these data showed that Putocrin® of 2.5% (v/v) did not exert adverse effect while exhibiting certain positive effects (increased loricrin content) on skin barrier.

**FIGURE 2:**
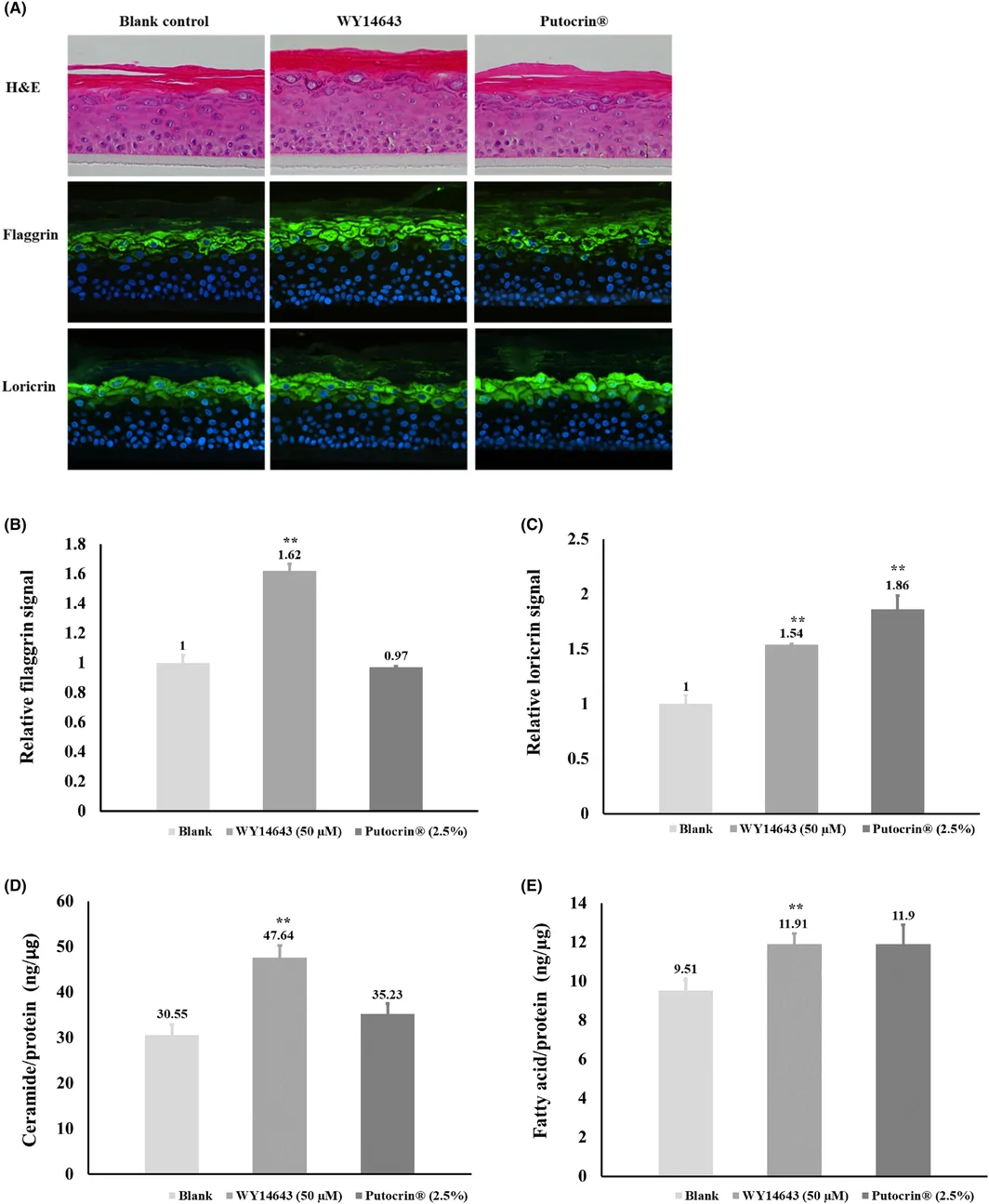
Effects of Putocrin® to EpiKutis® in vitro skin. EpiKutis® skin was pre‐incubated with 2.5% (v/v) Putocrin®, 50 μM WY14643 (positive control) and EpiGrowth culture medium (blank control) respectively for 24 h. Part of the skin was fixed, sectioned, and processed for hematoxylin/eosin (H&E) and filaggrin and loricrin immunostaining analysis, while the other portion of the skin was proteinase K digested to acquire the stratum corneum to analyze its lipid content using LC–MS. (A) Putocrin® did not affect skin morphology (H&E staining) and filaggrin (green fluorescence), but increased loricrin (green fluorescence). (B) Putocrin® did not affect filaggrin content. (C) Putocrin® increased loricrin content by 86.00% (*p* < 0.01). (D) Putocrin® did not change ceramide/protein ratio; (E) Putocrin® did not change fatty acid/protein ratio. Data were presented as mean ± SEM. “**” denotes compared with blank control, *p* < 0.01.

### 3.3 Putocrin® enhanced the penetration of Fitc‐labeled tranexamic acid into pigskin

The pigskin‐Franz cell system was used to investigate the skin penetration enhancing activity of Putocrin®. The experiments included a blank control group, 2% tranexamic acid group, 2% Fitc‐labeled tranexamic acid group, and Putocrin® with 2% Fitc‐labeled tranexamic acid group. Each group performed in triplicates. HPLC analysis of tranexamic acid and Fitc‐labeled tranexamic acid got a very good standard curve with *R* ^2^ of 0.99998 and 0.99914, respectively. HPLC quantification of tranexamic acid and Fitc‐labeled tranexamic acid in the PBS receiving solution at 1, 6, and 24 h posttreatment (Figure 3A) showed that tranexamic acid efficiently penetrated the skin and reached the receiving solution within 1 h with a cumulative permeability of 5.095 ± 0.250 mg. Fitc‐labeled tranexamic acid could barely penetrate the skin and its presence in the receiving solution could only be detected at 24 h post exposure. With the presence of Putocrin®, Fitc‐labeled tranexamic acid penetration efficacy was significantly increased and could be detected at 1 h post exposure and with a cumulative permeability of 4.943 ± 0.273 mg. Further calculation of the percentage of diffusion (Figure 3B) revealed that Putocrin® increased the skin penetration of Fitc‐labeled tranexamic acid from 0.00% to 10.014% within 1 h and from 1.121% to 83.983% within 24 h, which was close to the tranexamic acid (90.013%). Linear regression analysis of cumulative permeability against penetration time found the transdermal coefficient Kp (i.e., penetration rate) of 0.192 mg/h for Fitc‐labeled tranexamic acid in the presence of Putocrin®, was much higher than that of Fitc‐labeled tranexamic acid (0.003 mg/h), and even higher than unlabeled tranexamic acid (0.124 mg/h). Further analysis of the skin surface wash off solution showed that only 0.281 ± 0.016 mg Fitc‐labeled tranexamic acid remained on the skin surface for Putocrin® with 2% Fitc‐labeled tranexamic acid group. The result was close to that of the 2% tranexamic acid group (0.200 ± 0.095 mg), while most of the Fitc‐labeled tranexamic acid remained on the skin surface for the 2% Fitc‐labeled tranexamic acid group (4.320 ± 0.205 mg). An additional skin tissue analysis revealed that there were less Fitc‐labeled tranexamic acid remained in the skin for the Putocrin® with 2% Fitc‐labeled tranexamic acid group (0.382 ± 0.032 mg) than the 2% Fitc‐labeled tranexamic acid group (0.693 ± 0.047 mg), while only little (0.067 ± 0.010 mg) tranexamic acid remained in the skin tissue for the 2% tranexamic acid group. Similar results were also observed in the skin cryosections (Figure 3C,D). A 300 μL of sample was loaded onto each skin, the exact loading volume was calculated by deducting the weight of the sample before and after loading. The exact loading amount for tranexamic acid was calculated to be 5.562, 5.885, and 5.661 mg for Putocrin® with 2% Fitc‐labeled tranexamic acid group, 2% Fitc‐labeled tranexamic acid group, and 2% tranexamic acid group, respectively. The corresponding tranexamic acid on the surface of the skin, inside the skin and in the recovery solution summed up to be 5.598, 5.595, and 5.661 mg respectively, which resulted to a recovery rate of 95.229%, 91.313%, and 94.702%, respectively. All these data demonstrated the strong enhancing permeation efficacy of Putocrin®.

**FIGURE 3:**
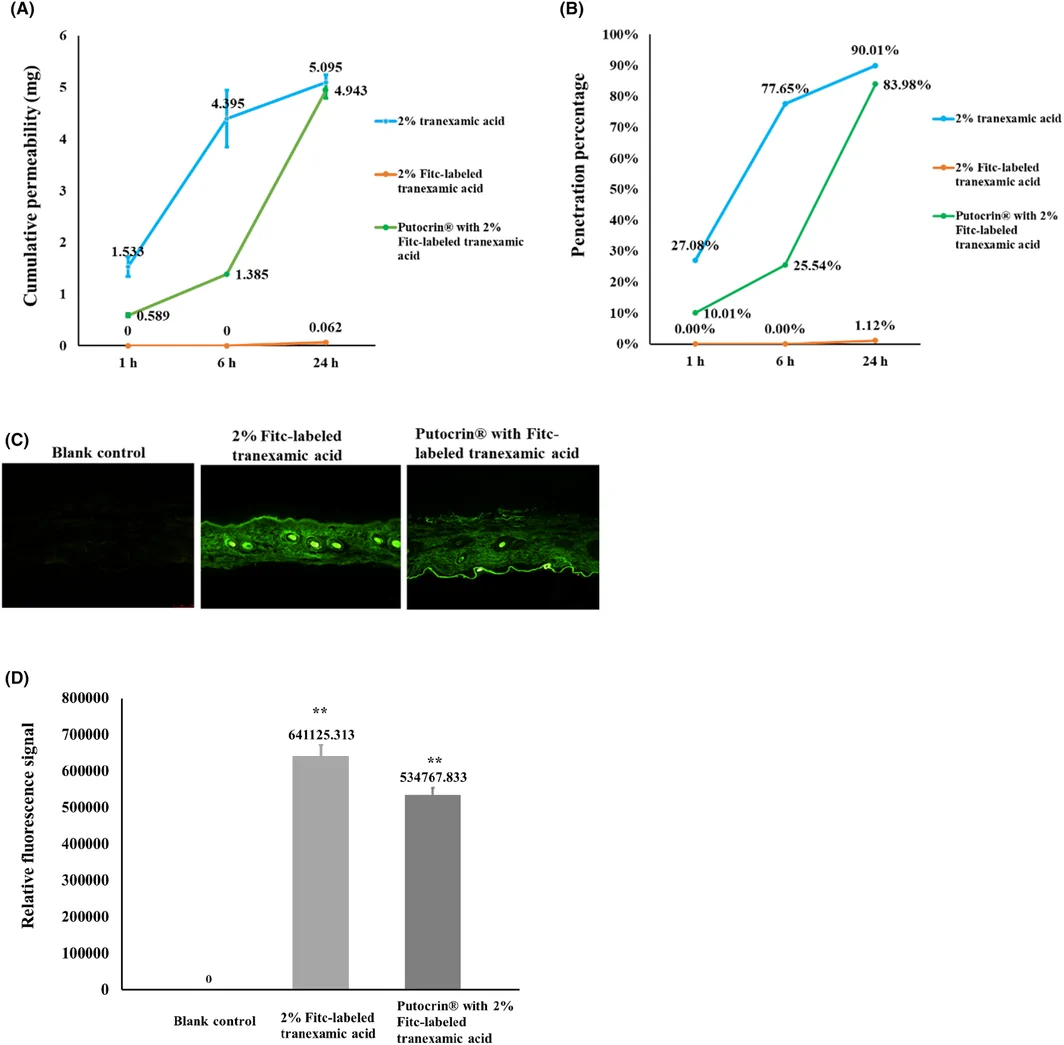
Enhancing penetration efficacy of Putocrin® to Fitc‐labeled tranexamic acid into pig skin. Pig skin in a Franz diffusion cell system was treated with Putocrin® and 2% Fitc‐labeled tranexamic acid, 2% Fitc‐labeled tranexamic acid, 2% tranexamic acid and PBS (blank control) respectively for 24 h. The cumulative permeability (A) and penetration percentages (B) of tranexamic acid/Fitc‐labeled tranexamic acid at 1 h, 6 h, and 24 h in PBS receiving solution was quantified by HPLC, and the Fitc‐labeled tranexamic acid remaining in pig skin were visualized through cryosection (C) and its signal intensity was measured and presented in (D). Data were presented as mean ± SEM of three independent experiments. “**” denotes compared with blank control, *p* < 0.01.

### 3.4 Putocrin® enhanced the penetration of Fitc‐labeled tranexamic acid into zebrafish embryos

The zebrafish embryo study results showed that 2.5% Putocrin® exerted no observable toxicity to zebrafish embryos, which was reflected by the last perfect fluorescence tail photo of Figure 4. Figure 4 also showed that embryos treated with 2.5% Putocrin® or 1% Fitc‐labeled tranexamic acid gave off no green fluorescence signal, while embryos treated with 2.5% Putocrin® together with 1% Fitc‐labeled tranexamic acid gave off strong green fluorescence, demonstrating the enhancing skin penetration activity of Putocrin® to Fitc‐labeled tranexamic acid.

**FIGURE 4:**
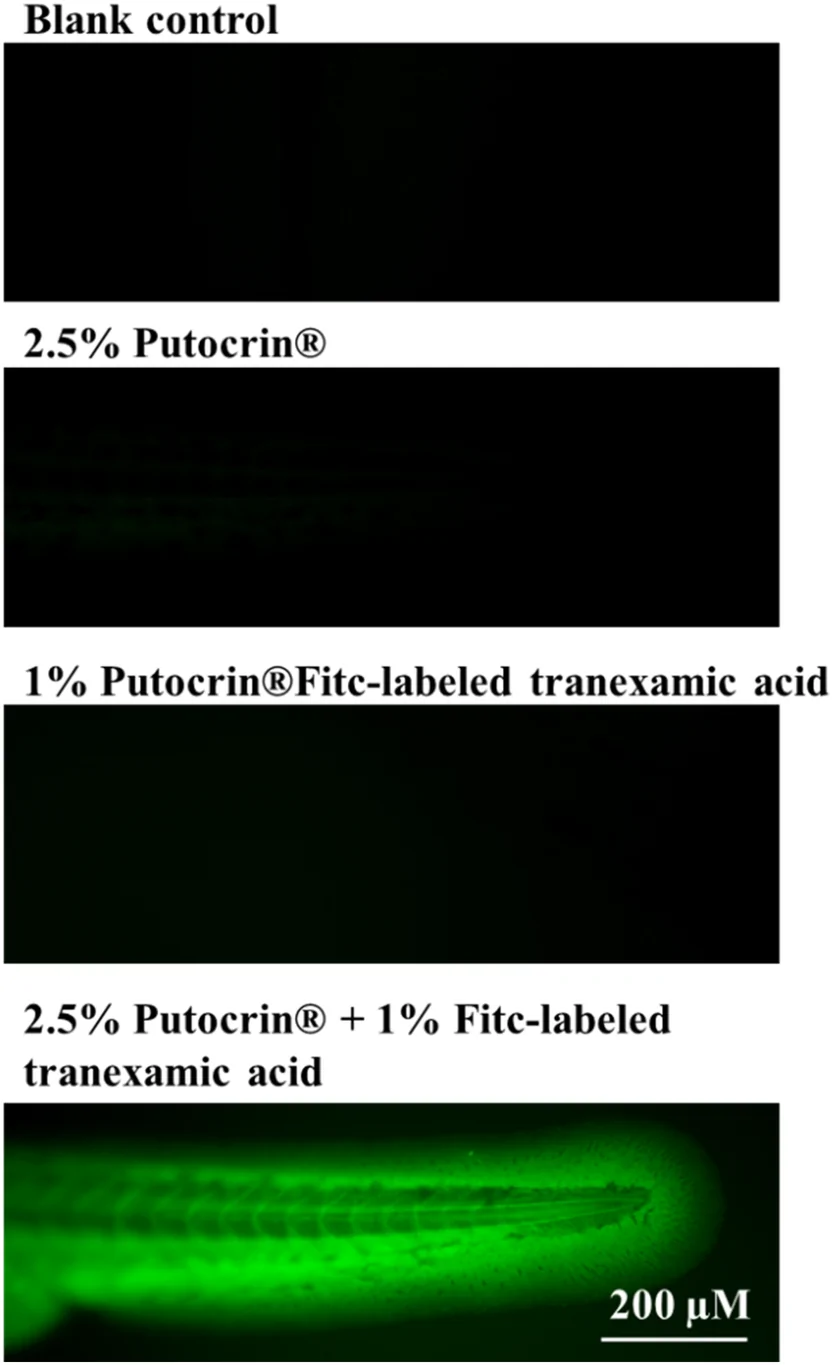
Enhancing penetration efficacy of Putocrin® to zebrafish embryos. Zebrafish embryos of 48 hpf were treated with 2.5% Putocrin®, 1% Fitc‐labeled tranexamic acid, 2.5% Putocrin® together with 1% Fitc‐labeled tranexamic acid, and water (blank control) respectively for 2 h, and the tail region of embryos were fluorescence imaged.

## 4 DISCUSSION

Active ingredient delivery through the skin remains a challenging pursuit for both pharmaceutical and formulation scientists. The stratum corneum is the superficial layer of our skin, contributes to the major barrier of cosmetics penetration. The stratum corneum mainly consists of cornified enveloped corneocytes and lipid matrix forming a brink‐and‐mortar structure. Loricrin that comprising more than 70% of the cornified envelope, contributes to the protective barrier function of the stratum corneum,^18^ the deduced expression of loricrin was reported to cause fragile barrier against mechanical stress.^19^ Filaggrin is another key protein in epidermal barrier, the reduction or loss of which results in a compromised skin barrier with disturbed lipid organization and a significant increase of free fatty acids.^20^, ^21^ Besides the proteins that are important to maintain strong “bricks,” skin lipids as “mortar” are also one of the most important compounds in maintaining barrier function in the stratum corneum that protects the body from desiccation.^1^, ^22^ The “mortar” is composed of ceramide, free fatty acid and cholesterol at a molar weight of 1:1:1 combination, and alteration of the skin lipid composition is known to impair the barrier function. For example, ceramide is the dominant barrier lipid by weight (over 50%), its content decrease will lead to barrier dysfunction such as an increase of trans epidermal water loss (TEWL) and decrease of protection against infection^23^; free fatty acids are another important species for skin barrier function and its composition alteration will also cause skin barrier dysfunctions such as microbiome disorder and inflammation.^22^ An ideal SPE should possess high permeation‐enhancing potency while exerting no adverse effects to the skin barrier.

In this study, the safety of SPE combination Putocrin® was investigated using a keratinocyte MTT assay and an in vitro EpiKutis® skin study. The suitability of the EpiKutis® for skin toxicity assessment have been reported.^24^, ^25^ The EpiKutis® was reported to meet the OECD TG 439 standard,^25^ and comparison study with EpiSkin™ showed that both were suitable for the OECD TG 439 skin irritation and OECD TG 431 skin corrosion.^24^ In this study, the keratinocyte assay showed that Putocrin® with a concentration no higher than 2.5% (v/v) exerted no obvious effect on cell viability. The in vitro skin experiment examined the effects of 2.5% (v/v) Putocrin® on the skin barrier and found out that it did not affect the skin morphology, the filaggrin protein content, ceramide/protein ratio or free fatty acid/protein ratios, while significantly increasing loricrin content by 86.00% (*p* < 0.01). As the major function of loricrin protein is to reinforce the stratum corneum and to enhance its defensive barrier function,^18^ the enhance loricrin synthesis activity of Putocrin® can be regarded as a positive effect of Putocrin® to the skin barrier.

After confirming Putocrin® is safe to the skin barrier, its enhancing skin penetration activity was examined using a pigskin‐Franz diffusion cell system following OECD TG 428.^8^ In this experiment, tranexamic acid with a molecular weight of 157.21 g/mol, penetrated the pig skin rapidly, Fitc‐labeled tranexamic acid with molecular weight of 546.59 g/mol, which is over 500 g/mol, barely penetrated the skin with low penetration rate of 1.121% at the end of the 24 h exposure. However, with the assistance of 2.5% (v/v) Putocrin®, the penetration of Fitc‐labeled tranexamic acid significantly increased to 83.983% within 24 h, which was close to the tranexamic acid (90.013%). This experiment demonstrated the strong skin penetration enhancing activity of Putocrin®.

The safety and enhancing skin penetration efficacy of Putocrin® demonstrated in vitro were further confirmed through an in vivo experiment using zebrafish embryos. The results showed that Putocrin® at 2.5% exerted no observable adverse effect to zebrafish embryos, indicating it is safe in vivo. Fluorescence microscope observation revealed that the Fitc‐labeled tranexamic acid could not penetrate through the skin of zebrafish embryos, while obvious fluorescence signal of the Fitc‐labeled tranexamic acid was observed within embryos with the presence of Putocrin®, which further confirmed the enhancing skin penetration activity of Putocrin® found in the pigskin‐Franz cell experiment.

In the Putocrin® combination, isosorbide dimethyl ether is a common solvent and SPE, its enhancing penetration activity was presented in a recent study in which it was selected by a computer‐aided formulation program named Formulating for Efficacy® to enhance the delivery of caffeine in eye cream.^26^ 1,2‐Pentanediol, a multifunctional moisturizer was reported to enhance the topical bioavailability of both hydrophilic and amphiphilic active ingredients in topical formulations.^27^ Inositol, a main component of the skin beneficial fatty acid lecithin was reported to significantly improve psoriasis when formulated with 40% urea.^28^ The present report is the first study to combine the three ingredients as SPE Putocrin®, and demonstrate that Putocrin® was safe to the skin barrier and it exerts strong enhancing penetration activity to Fitc‐labeled tranexamic acid.

## 5 CONCLUSION

The importance to formulate a drug or cosmetics which can efficiently deliver active ingredients through the epidermis while causing no adverse effects to the skin barrier is essential for SPEs characterization. This study combined an in vitro keratinocyte assay, in vitro EpiKutis® skin study, and pigskin‐Franz diffusion cell experiment to demonstrate the safety and remarkable enhancing skin penetration efficacy of the SPE combination Putocrin®, which is now being applied to cosmetics formulation.

## AUTHOR CONTRIBUTIONS

ZC and ZC designed, coordinated the study and proofread the paper; DW and JC performed and analyzed the experiments; XC designed, analyzed, and wrote the paper. All authors reviewed the results and approved the final version of the manuscript.

## CONFLICT OF INTEREST STATEMENT

The authors declare that they have no known competing financial interests or personal relationships that could have appeared to influence the work reported in this paper.

## ETHICS STATEMENT

This manuscript did not include any human subjects and animal experiments.

## Supporting information


Figure S1.

## Data Availability

Research data are not shared.
