# Supplementary material for: Evaluation of the safety and efficacy of skin penetration enhancer Putocrin®
Source: J Cosmet Dermatol. 2024 Jun 12;23(10):3272–80. doi: 10.1111/jocd.16409 (PMC13648913; doi:10.1111/jocd.16409)
Supplement: Supplementary file 1 [file JOCD-23--s001.docx]

Suppl fig 1. Diagram of suckling pigskin-Franz diffusion cell system. The structure of TK-12D type transdermal diffusion tester includes a constant temperature water bath, a control box, an operating table, a transdermal diffusion cell, a rehydration tube and a connecting tube. The upper part of the inner bottle of the transdermal diffusion cell was provided with a control box for the refill port, which was connected with the constant temperature water bath and the operating table through electric wires respectively. The tube was communicated with the rehydration port of the transdermal diffusion cell, and the transdermal diffusion cell was placed on the operating table. The device could carry out multiple transdermal experiments at the same time without interfering with each other and without bubbles. The volume of the receiving chamber was 8.5mL and the diffusion area was 3cm^2^. The diffusion cell was composed of upper and lower cylindrical glass tubes, and the patch sandwiched between them divides the upper and lower chambers into two chambers. The upper chamber was a diffusion chamber, the lower chamber was a receiving chamber, and a sampling tube was connected to the right part of the receiving chamber for sample injection, sampling, and bubble discharge.
